# Unsupervised learning reveals novel disease-associated proteins in high-dimensional human proteomic data

**DOI:** 10.1038/s41598-026-41385-7

**Published:** 2026-02-22

**Authors:** Elvis Bernard, Yiling Wang, Manlin Chen, Shunqing Xu

**Affiliations:** 1 School of Environmental Science and Engineering, Hainan University, Haikou, 570228 China; 2 School of Life and Health Sciences, Hainan University, Haikou, 570228 China

**Keywords:** Biomarkers, Cancer, Computational biology and bioinformatics, Diseases

## Abstract

Modern advancements in precision medicine have led to the generation of vast proteomic datasets, capturing the concentrations of thousands of proteins across tens of thousands of participants. These datasets are traditionally processed using supervised learning methods due to their relative simplicity to implement and assess the output. However, this approach can sometimes overlook subtle patterns that might offer deeper insights. In contrast, unsupervised learning, while capable of revealing hidden relationships, struggles with the challenge of high dimensionality, meaning that brute-force analysis could take millennia to complete. In this study, we developed the Dimensionality Reduction with Avoidance of Missing/COmmunity Detection (DIRAM/COD) framework to address this problem by combining dimensionality reduction techniques with unsupervised learning to analyze the massive proteomic dataset of the UK Biobank, which includes the concentrations of 2,923 plasma proteins from 52,691 participants. By applying this novel approach, we not only confirmed well-established biomarkers for diseases such as hypertension (UBE2L6) and leukemia (LRCH4) but also identified novel protein candidates. For instance, we identified IGF2BP3 in connection with celiac disease, a protein previously linked to intestinal barrier function, along with several other proteins not yet associated with these diseases. This approach opens up exciting possibilities for future research and may pave the way for the discovery of new biomarkers and therapeutic targets.

**Supplementary Information:**

The online version contains supplementary material available at 10.1038/s41598-026-41385-7.

## Introduction

Proteins circulating in the human bloodstream provide critical insights into an individual’s health status^1^. These plasma proteins play essential roles in cell signaling, transport, growth, repair, and immune defense^2^ and include proteins released from damaged cells^3^. The human blood proteome offers a comprehensive snapshot of an individual’s health by capturing the interactions between thousands of circulating molecules^4^. These interactions reflect the combined influences of genetics, lifestyle, environment, comorbidities, and medications^5–7^.

Given the dynamic nature of the plasma proteome and the accessibility of blood sampling, these proteins serve as invaluable tools for diagnosing and predicting diseases^8^, identifying therapeutic targets^9^, and understanding disease mechanisms^10^. Proteomics has led to significant discoveries in gene‒protein interactions^11^, disease biomarkers^12^, aging^13^, and pharmacology^14^; however, its potential for systematically studying and assessing the risk of multiple future health conditions remains largely underexplored.

Historically, most predictive research using proteomics has been performed through supervised learning, relying on case-control studies^15^ to compare the plasma proteomes of healthy individuals and those diagnosed with diseases such as dementia^16^, Alzheimer’s^17^, coronary heart disease^18^, and type 1 diabetes^19^. Additionally, although shared molecular pathways have been identified in closely related diseases, limited knowledge exists concerning potential common mechanisms linking seemingly unrelated conditions. This gap highlights the need for a more systematic approach to understanding disease progression in humans.

The complexity of proteomic data presents both opportunities and challenges^20,21^. High-dimensional datasets contain vast amounts of information that can reveal intricate patterns of biological variation^22,23^. However, extracting meaningful insights from these data is difficult, particularly when the underlying structure is not immediately apparent^24^. Unsupervised learning algorithms excel at identifying hidden structures in high-dimensional datasets^25^. This capability makes them particularly useful in proteomics^26^, where they can detect subgroups of individuals with similar protein expression profiles^27^. By segmenting populations based on the similarity of their proteomic profiles, unsupervised learning can help uncover novel biological subtypes or identify biomarkers for disease stratification without prior knowledge of specific disease classifications or biological pathways^28,29^.

Here, using the UK Biobank Proteomic dataset containing the concentrations of 2,923 plasmatic proteins from 52,691 participants, we explored the potential of proteomic segmentation clustering together individuals with similar health profiles. We used two different methods, one based on density-based clustering, which we named Dimensionality Reduction with Avoidance of Missing (DIRAM), and one based on community detection, Dimensionality Reduction with COmmunity Detection (DIRCOD), to create different clusters^30,31^. We showed that the clusters created using these two methods were biologically meaningful and can provide new insights into the biology of different diseases because of the unbiased nature of the unsupervised machine learning methods. With respect to the three diseases we chose to analyze in more depth, we were able to highlight known actors while also bringing attention to unknown but plausible actors. Finally, we compared the advantages and disadvantages of these two different methods, with DIRAM delivering more compact clusters and DIRCOD creating larger clusters that are more suitable for data analysis.

## Results

### Unsupervised analysis of a large population

**Fig. 1:**
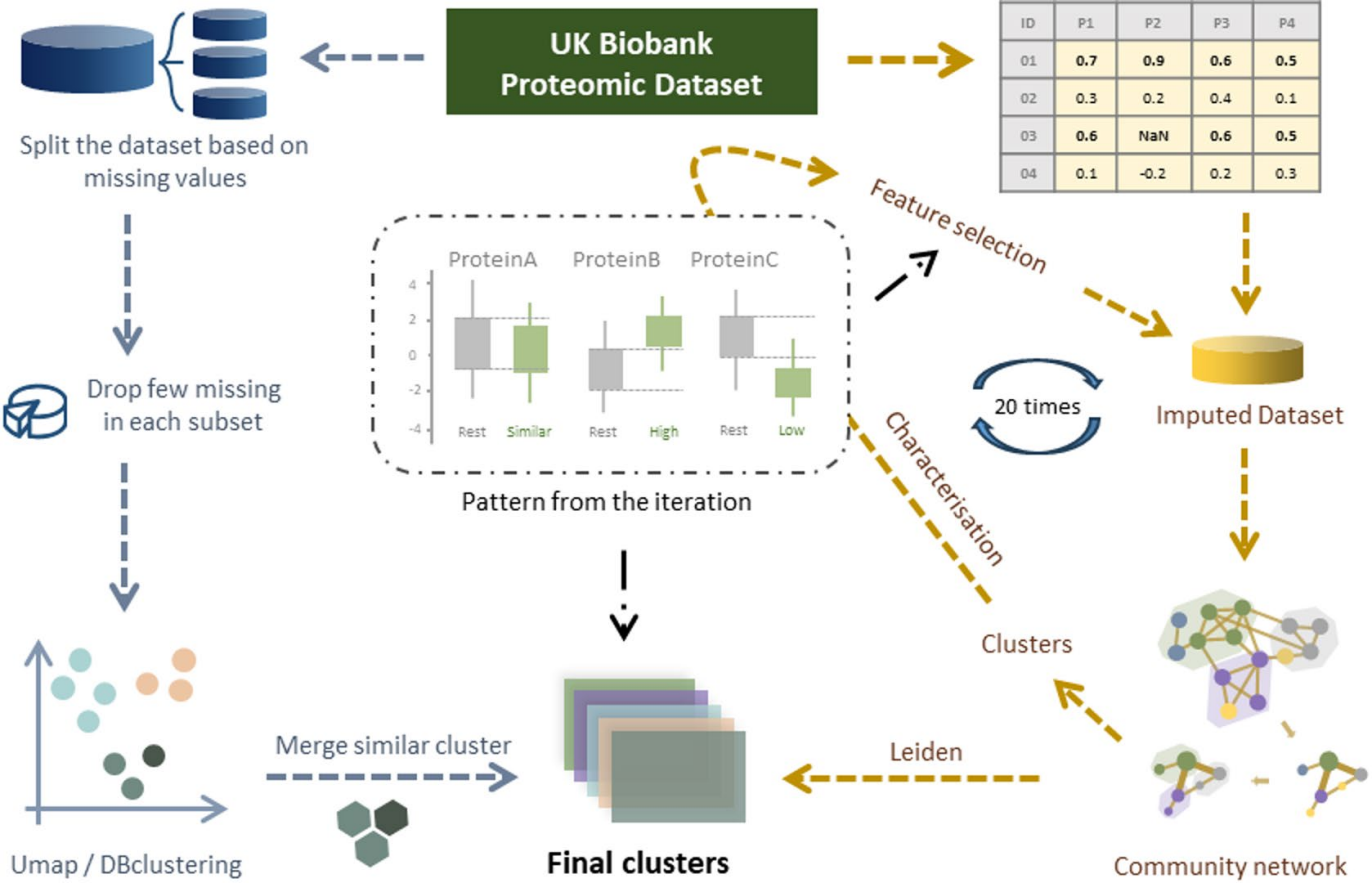
Schematic of the DIRAM/COD framework for the creation of the clusters. The blue path on the left represents the DIRAM method, where the dataset was sliced to avoid missing values. The yellow path on the right represents the DIRCOD method, where the Leiden community algorithm was used. The central medallion illustrates our definition of high- and low-abundance proteins.

The two primary challenges in identifying patterns in high-dimensional real-world data are missing values and the large number of dimensions^32^. Each additional dimension increases data sparsity, making patterns more difficult to detect. Imputation is a common strategy for handling missing data^33^; however, in large-scale proteomic datasets, missing data often reflect technical detection limits or biological variability, making reliable imputation infeasible. To mitigate both challenges, we developed two distinct clustering workflows (see Fig. 1).

The first approach, DIRAM, focused on avoiding missing values. During our initial analysis, we observed that certain groups of protein expression data shared missing values for the same participants. In these groups, removing protein expression data with missing values did not result in any loss of information. For the remaining variables, we grouped them based on the correlation of their missing values, minimizing the impact of removing incomplete observations. Each of these subdatasets was then processed through the same workflow: (1) dimensionality reduction to two dimensions using UMAP projection^34^; (2) cluster detection using the density-based clustering algorithm DBSCAN^35^, which allows the detection of an unspecified number of clusters of arbitrary shape in the presence of noise; and (3) merging clusters from different subdatasets that contained similar observations. This process was applied three times: once for the first release of proteomic data (8 clusters), once for the second release (13 clusters), and once using the combined dataset from both releases (16 clusters). As a result of this approach, 37 clusters have been defined.

The second approach, DIRCOD, aimed to reduce the impact of extra dimensions. This strategy involved two main steps. First, we applied the Leiden community detection algorithm to the dataset imputed using KNN^36,37^. KNN is a very common method of imputation that has already been used successfully by Jia You et al. on half of the UK Biobank proteomic data^8^. The detected communities were subsequently analyzed in the full raw dataset (non-imputed without any filtering) to identify protein expression variables (dimensions) that exhibited significantly different distributions within each community compared with the rest of the population. These distinct dimensions were then reintroduced into the first step. This iterative process was repeated 50 times without any sign of convergence. As a result, the first 20 iterations were used to assign observations to clusters based on patterns appearing in these 20 community detection results. As a result of this approach, 18 additional clusters have been defined, resulting in a total of 55 clusters.

### Characteristics of the unsupervised population clusters

In a first attempt to characterize the participants in each cluster, we looked at the age, sex and expression of the different proteins for each cluster (see Tables S1–S2–S3). The age distribution of 14 clusters significantly differed from that of the whole population; among them, the age distribution of 6 clusters exhibited a p value lower than 10^− 5^ (see Table S1). Most clusters had a balanced population in terms of sex, while for 19 clusters, one sex represented less than 40% of the population (see Table S2). All the clusters varied greatly in terms of protein concentration. We decided to take a very conservative approach where for a protein to be considered to be present at a different concentration, the concentrations in the top 75% of the participants in this group should be higher than the concentrations in the bottom 75% of the participants in the other group (see Table S3).

To better describe the participants in the different clusters, we used the ICD10 code of the diagnosis linked to each participant (see Table S4). Remarkably, the results of the analysis provided different insights: both clustering methods can identify similar clusters, as shown in Clusters A8 and B9, both of which contain a high proportion of very sick people (organ failure, transplant, cancer, etc.). Interestingly, compared with Clusters B5 and B6, which had a higher prevalence of hypertension and were characterized by higher protein concentrations, Clusters B1 and B2 had a low prevalence of hypertension-related diseases and lower concentrations of a set of proteins.

We then conducted a GWAS for each of the clusters. The most significant associations are reported in Table S5. While it is clear that some variants were positively selected in different clusters, as reflected by their extremely low associated p value, their relationship with the observed phenotype was not clear (see Fig. S1).

We finally evaluated the questionnaire data, medication prescription and blood chemistry. We did not find any significant associations between these data and the different clusters. Surprisingly, we found that the medication prescription records were most likely incomplete. Although we were expecting to find some strong medication prescribed to the participants from Clusters A8 and B9, as these participants suffer very severe disease (e.g., organ transplant, cancer, or amputation), we did not find any of these associations.

### Identification of disease-related clusters

**Fig. 2:**
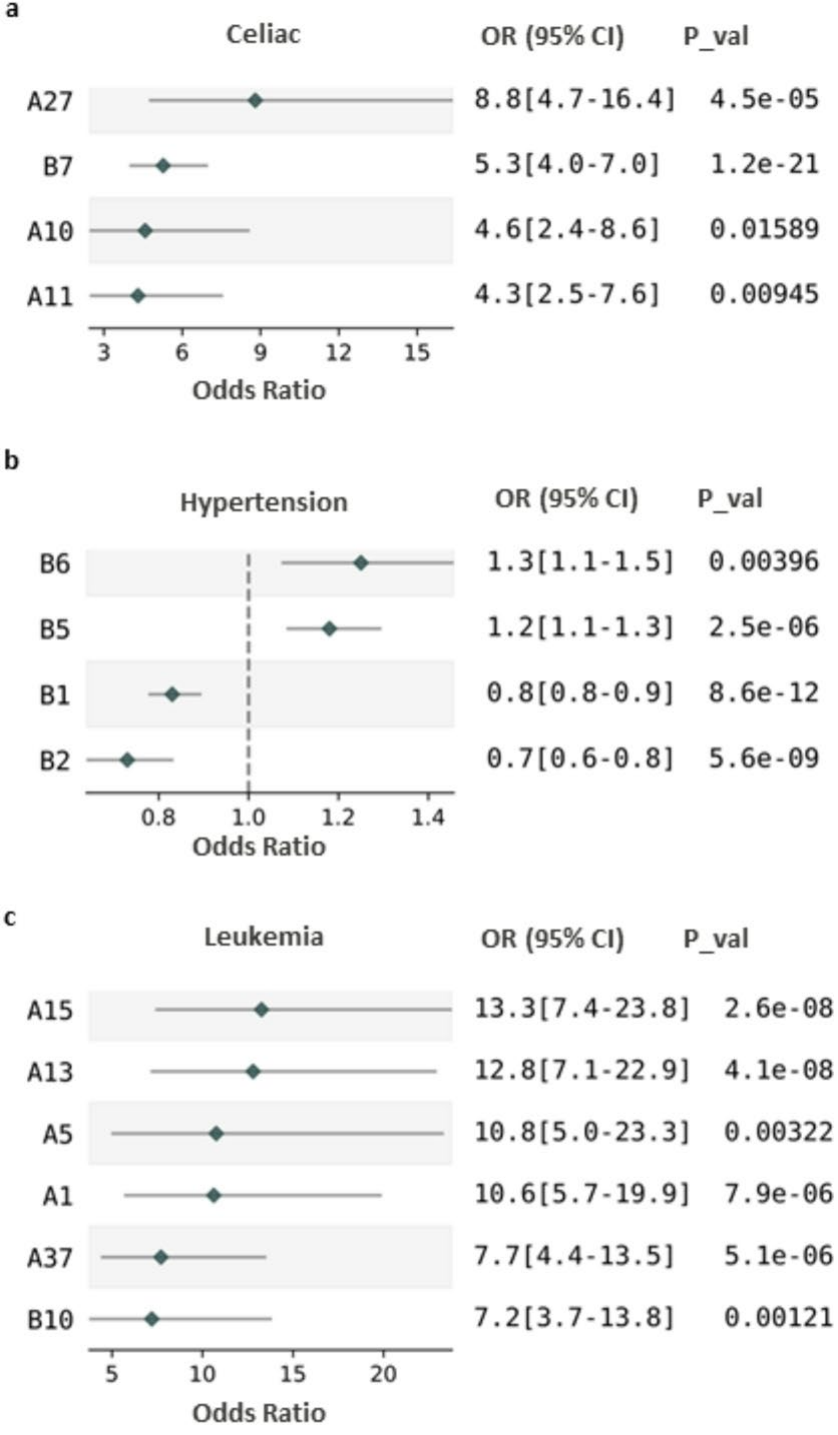
Odds ratios. Representation of the odds ratios for the different diseases studied and the clusters considered. (**A**) Celiac disease. (**B**) Hypertension. (**C**) Leukemia. OR: odds ratio, CI: confidence interval.

We next wanted to investigate whether any biological insights could be found from these groups of clusters (see Fig. 2). For the first time, we looked at the proteins that were common between the clusters with the same disease. We chose to focus our attention on celiac disease (ICD 10 code: K90), hypertension (ICD 10 Code: I10) and leukemia (ICD10 code: C91). For each of these diseases, we identified several common proteins with high or low abundance (see Table S6).

**Fig. 3:**
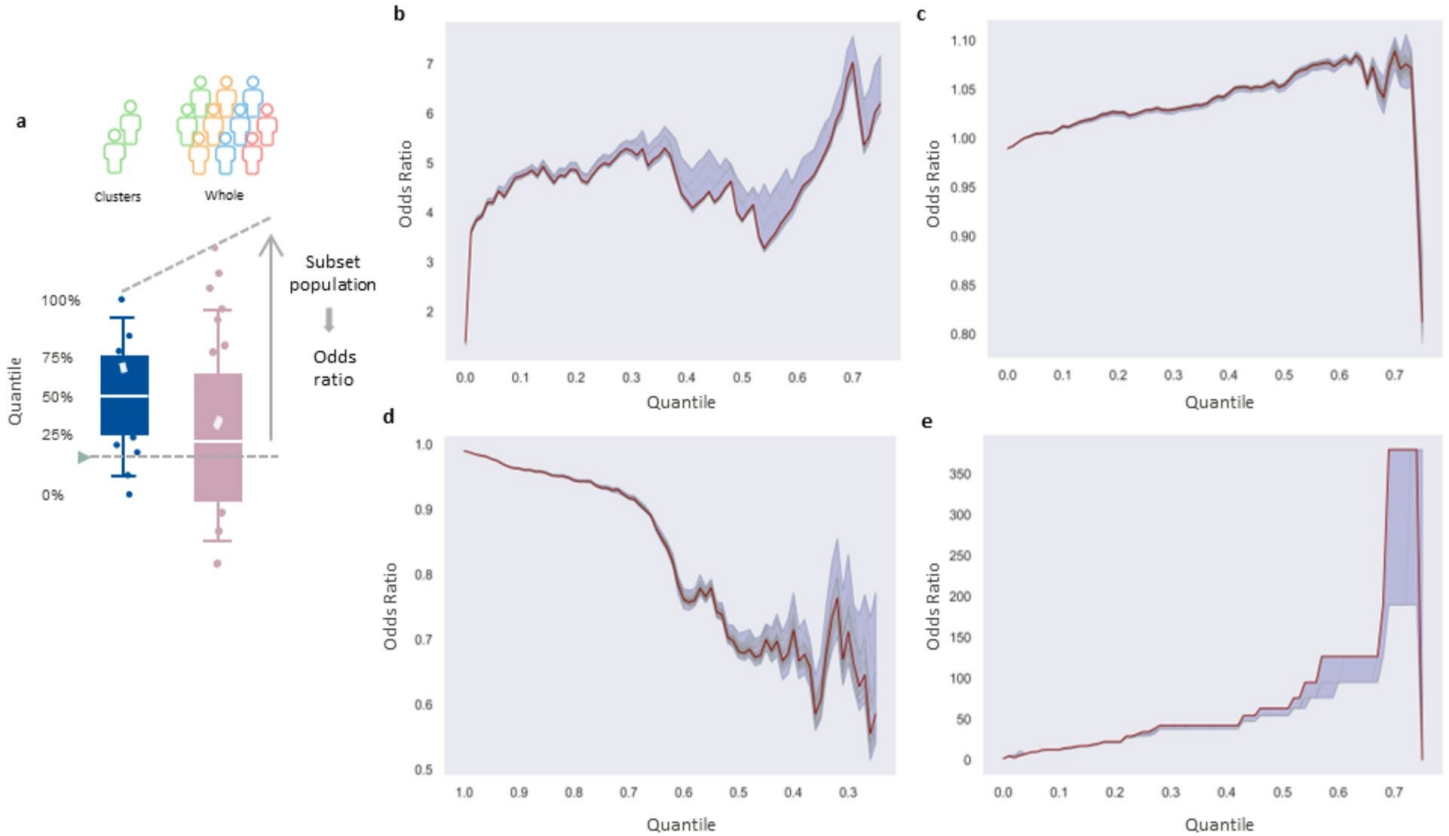
Recreation of the clusters. (**A**) Representation of the slicing. Blue: cluster of interest; Red: whole population. The quantiles have been defined on the cluster of interest, and the value has been used as a cutoff value for the whole population. (**B**) Celiac disease. (**C**) Abundant proteins in hypertension. (**D**) Rare proteins in hypertension. (**E**) Leukemia. The red line represents the odds ratio of the cluster when all the proteins were used; the green line represents the odds ratio of the cluster when all but one protein were used.

To assess whether these proteins could reflect the identity of the disease cluster, we tried to create disease-rich clusters based on these proteins. First, we regrouped all the participants of the original clusters into a single cluster; then, for highly abundant proteins, we sliced the full population above a threshold corresponding to the different percentiles in the single cluster, and for the proteins with low abundance, we looked at the population below the threshold (see Fig. 3a). Using this method, we were able to create clusters with significantly higher or lower odds ratios than the original population. For celiac disease, we were able to create clusters with odd ratios of 5 or higher (see Fig. 3b). For hypertension, if we only looked at the common proteins that were differentially distributed in Clusters B1, B2, B5 and B6 for this analysis (77 proteins), we reached an odds ratio of 1.05 or higher for the overly abundant proteins and 0.8 or lower for the less abundant proteins (see Fig. 3c and d). We were also able to reach an odds ratio of 0.9 or lower using only the proteins with low abundance in the case of Cluster B15 for hypertension (see Fig. S2a and b). Finally, the same analysis was performed in the case of leukemia, and we were able to reach an odds ratio of 50 or higher, but the lower number of participants diagnosed with leukemia affected the robustness of the analysis (see Fig. 3e).

We then used this approach slightly differently by removing one protein for each of the analyses. If the missing protein has no impact on the disease, then removing it from the analysis will improve the result, but if the protein is important for the disease, then the result will appear worse. In the early iterations of the analysis, we used the Benjamin–Hochberg correction for the p value, and Cluster A7 was included in the analysis for hypertension; we noticed that removing ube2l6 from the analysis had a dramatic effect on the odds ratio, decreasing it by 0.01 (from 1.036 to 1.026) (see Fig. S2c and d). When we moved from the Benjamin–Hochberg correction to the Bonferroni correction, we had to remove Cluster A7, and the dramatic effect of ube2l6 was lost; however, removing it from the analysis still had a strong effect, indicating its importance for hypertension. In addition to ube2l6, hnrnpul1 and becn1 had similar effects (see Table S7). These 3 proteins have already been shown as being involved in hypertension or related disease^38–40^. Similar analysis has been conducted on the cluster with celiac disease and igf2bp3 was the protein having the strongest effect. This protein has been shown to be involved in the intestinal barrier function^41^. Other proteins with strong effect are nrxn3 and cacnb1(see table S7).

**Fig. 4:**
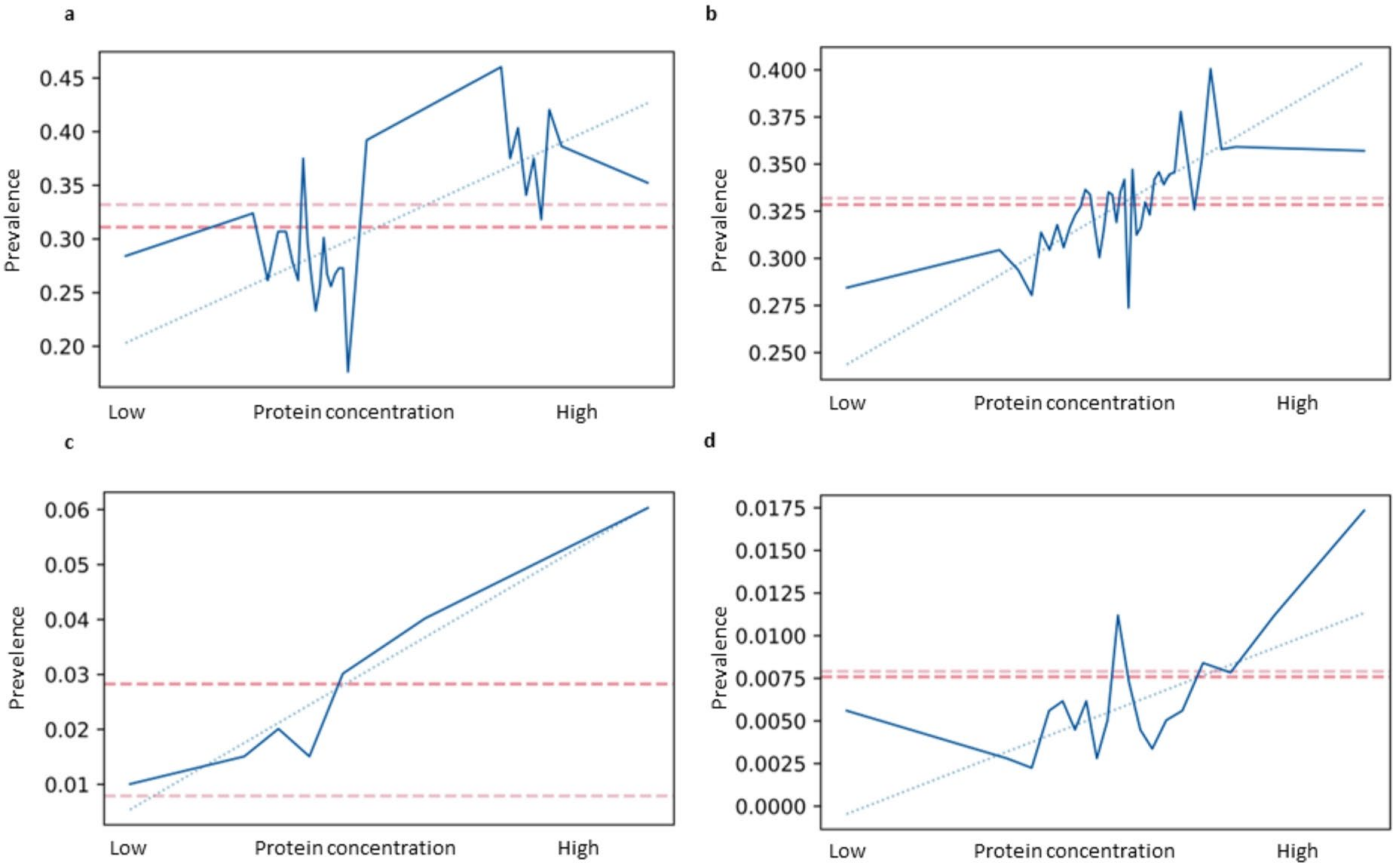
Prevalence of the disease along an artificial axis. (**A**) First axis of a PCA transformation on the group of clusters for hypertension. (**B**) First axis of the same PCA transformation applied to the full population. (**C**) First axis of a PCA transformation on the group of clusters for celiac disease. (**D**) First axis of the same PCA transformation applied to the full population. Red dashed line: prevalence in the cluster used for the PCA; pink dashed line: prevalence in the whole population; blue dotted line: linear regression.

As the of different common proteins seem to be linked to the probability of having a disease, we then tried to combine the different protein concentrations into one unique value by looking at the first dimension of a PCA reduction^42^. We first applied this approach to the case of hypertension where clusters with opposite values were available. As shown in Fig. 4a, the first dimension of the PCA seemed to capture the relationship between the concentration of the proteins and the prevalence of the disease for the participants in the clusters used. When applied to the full population, the relationship between the first dimension of the same PCA reduction and the incidence of hypertension was conserved, indicating that the relationship was not specific to the population used in the PCA (see Fig. 4b). We performed the same analysis for celiac disease and found similar results (see Fig. 4c and d). Unfortunately (from a data analytical point of view), owing to the low number of participants suffering from leukemia, we were not able to conduct the same analysis.

**Fig. 5:**
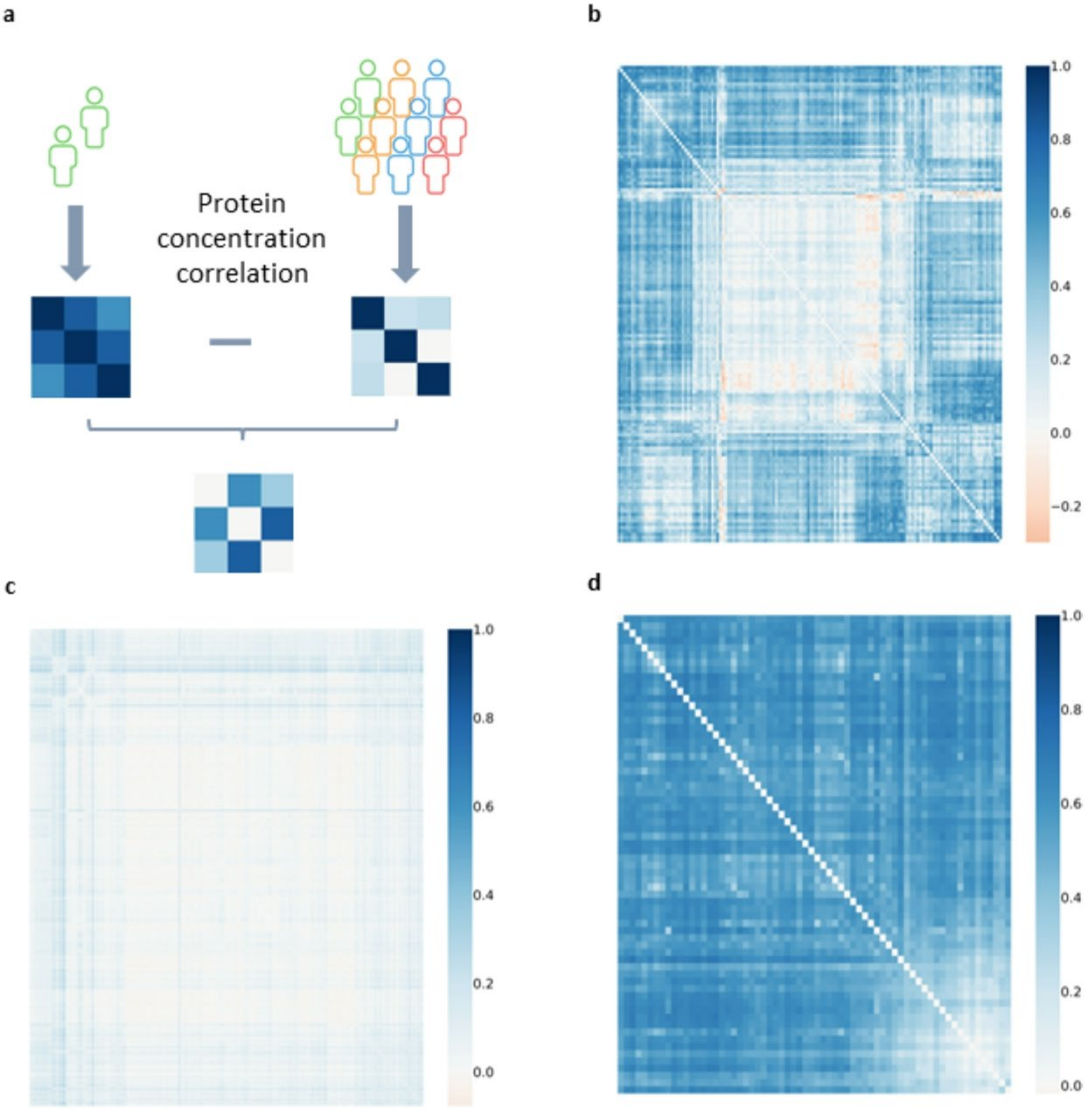
Differences in correlations between the proteins. (**A**) Schematic of the analysis. Difference between the correlation matrix for the differentially distributed proteins for the population in the cluster versus the rest of the population. (**B**) Leukemia. (**C**) Hypertension. (**D**) Celiac disease.

In an attempt to better understand the clusters with a higher prevalence of leukemia, we looked at the correlation between the different proteins differentially expressed in these clusters and compared them with those in the rest of the population (see Fig. 5a). These results revealed few proteins whose correlations between the participants in our cluster were very similar to their correlations in the rest of the population (see Fig. 5b). The correlations of some pairs of proteins decreased by almost 0.3, indicating a loss of coregulation, whereas those of most of the proteins increased by up to 0.7, indicating stronger coregulation of these proteins in these participants and suggesting a potential role in leukemia (a correlation of 0.16 was significant for a sample of that size after Bonferroni correction for each pair of proteins tested). Interestingly, the main proteins whose expression levels decreased were lrch4, wdr46, serpinb1 and nub1. Lrch4 has been associated with leukemia^43^, and WDR46 has been associated with the development of gastric carcinoma, colorectal cancer and hepatocarcinogenesis^44^; the serpine family is known to be involved in cancer, and nub1 has been identified as a biomarker in cancer^45,46^.

The same analysis in the case of hypertension revealed less dramatic results, with only a decrease of − 0.05 and an increase of 0.12, which is in accordance with the view that hypertension is a widely multifactorial health problem (significant correlation after Bonferroni correction for that sample: 0.05) (see Fig. 5c). In the case of the clusters representative of celiac disease, we did not observe any significant decrease in correlation, but we observed a strong increase of 0.7 in correlation for almost all the proteins considered (significant correlation after Bonferroni correction for that sample: 0.09) (see Fig. 5d). These results clearly indicated that some changes in the coregulation of different proteins occur in leukemia and celiac disease.

## Discussion

Plasma protein cohort datasets have primarily been analyzed using supervised machine learning approaches, and multiple predictive models have already been developed based on these data^8,16,47,48^. The strong interest in plasma protein datasets underscores their significant promise for research discovery. However, to the best of our knowledge, these datasets have not yet been explored using unsupervised machine learning methods.

This research was designed to explore how unsupervised machine learning can be used to extract meaningful insights from large proteomic datasets. These datasets often contain the concentrations of thousands of proteins, and sifting through all possible combinations to find patterns is an incredibly time-consuming task. In fact, owing to the sheer volume of potential combinations (2 ^number of proteins^), such an exhaustive search could take longer than a human lifetime, even with thousands of iterations performed every second. In this study, we evaluated the effectiveness of the DIRAM/COD framework using two different machine learning techniques to segment the UK Biobank proteomic data into distinct clusters that have biological significance. Our goal was to identify ways in which these methods could help reveal patterns within the data that are both insightful and actionable for future research.

Although both methods aim to reveal the same underlying biological patterns, they result in clusters of vastly different sizes. In the DIRAM method, where the dataset was divided to avoid missing data, we observed smaller clusters. Conversely, in the DIRCOD method, where missing data were imputed, the clusters were much larger. Although larger clusters benefit from a larger sample size, making them more likely to include individuals with rare diseases and thus offering more robust statistical power, they come with a trade-off: the imputed values may not represent the true biological reality but are instead estimates of the “best value”. To address this, we introduced a validation step using the nonimputed data, helping to minimize the bias introduced by imputation and ensuring that our findings are as accurate and reliable as possible.

To minimize false positives, we applied conservative Bonferroni corrections to the p values in this study. Although this approach ensures a high level of statistical rigor, it may not be ideal for researchers focused on more specific phenotypes or diseases, who could benefit from a less stringent correction. For instance, adjusting the p value correction affected the inclusion of Cluster A7 within the hypertension group, leading to different results. Similarly, Cluster A8, which initially had an uncorrected p value of 10⁻⁴ for celiac disease, had an increase in this value to 0.06 after correction. For more targeted studies, the Bonferroni correction may be too conservative, and researchers focused on specific conditions will likely find that less strict p value corrections are more appropriate for drawing meaningful conclusions without unnecessarily discarding relevant clusters.

DIRAM/COD successfully identified clusters with biological significance, providing valuable insights into a variety of diseases. For example, in the case of celiac disease, our analysis highlighted several key proteins, including IGF2BP3, NRXN3, LRP2BP, and CACNB1. IGF2BP3 has already been implicated in maintaining the intestinal barrier^41^, whereas LRP2BP is closely related to LRP1, which has been previously associated with celiac disease^49^. NRXN3, which is known to be active in the abdominal region^50^, has also emerged as an important candidate. In the case of leukemia, the analysis revealed the misregulation of proteins such as LRCH4, WDR46, SERPINB1, and NUB1. LRCH4 has been previously linked to leukemia^43^, and WDR46 has been associated with gastric carcinoma, colorectal cancer, and hepatocarcinogenesis^44^. SERPINE family members are well known for their involvement in cancer^45^, and NUB1 has been identified as a cancer biomarker^51^. With respect to hypertension, we identified UBE2L6, which has been implicated in this disease^38^, HNRNPUL1, for which some variants have been identified as risk factors for coronary heart disease^39^, and BECN1, which has been implicated in pulmonary hypertension^40^. In summary, our analysis not only reinforced the roles of well-established biomarkers but also identified potential new candidates, offering a fresh perspective on these diseases and their underlying biology.

GWAS revealed that PNLIPRP1 and PNLIPRP2 played major roles in the segmentation observed in the second method, with variants in these genes showing extremely low p values in association with several clusters. However, owing to the highly conservative approach used to determine significant protein concentration differences, proteins linked to these genes were largely excluded from the analysis, and their potential roles were not fully explored. For example, Clusters B1 and B2 presented high levels of PNLIPRP1, whereas Clusters B5 and B6 presented lower levels. These contrasting concentrations of PNLIPRP1 suggest a protective role for this protein in the context of hypertension, warranting further investigation.

Interestingly, Cluster B15 had a low incidence of hypertension despite having some characteristics of a cluster with a high incidence of hypertension (high levels of protein shown in Figs. 3 and S2 and the presence of similar SNPs as B5 and B6). This finding is in accordance with the view that hypertension is a multifaceted disease and that having some predisposing factors can be compensated by other protective factors.

Another interesting finding during the realization of the project involved Clusters B3 and B6. These clusters had odds ratios of 13.29 for hepatitis (K73) and 9.46 for disease of the spleen (D73), respectively, both among women. Although the number of incidences was too low to allow us to perform a thorough analysis, it is clear that these results were not just the fruit of coincidence, suggesting that some of the 420 common proteins that are differentially distributed could be involved in both diseases.

Two clusters that particularly attracted our attention were A8 and B9. Despite having a high number of severely ill participants, we were unable to pinpoint a clear reason behind these groupings. These clusters were characterized by a high incidence of complex, nontrivial diseases. For example, A8 had prevalence rates of 49.6% for acute renal failure, 38% for surgical procedures with later complications, and 25% for individuals with transplanted organs. The main common factor we initially considered was the potential use of heavy medication. However, a surprising finding from the drug prescription records revealed that these individuals did not appear to be prescribed strong medications or, in some cases, any medications at all. Given that we have ruled out all other available variables, we believe that the use of heavy medication may still be the most likely unifying factor behind these two clusters. This suggests that the UK Biobank’s drug prescription data may be incomplete, warranting further investigation.

In conclusion, this study demonstrates that unsupervised learning applied to proteomic datasets can provide valuable new insights into various diseases. The framework we have developed, DIRAM/COD, can help scientists achieve this goal. One key limitation we encountered was the relatively small number of participants with specific diseases. However, this issue is being actively addressed by the UK Biobank, which is expanding its dataset from 2,923 proteins in 50,000 participants to 5,400 proteins in 600,000 participants. With this significant increase in data, we believe that this approach will uncover unexpected insights into the biology of rarer diseases, offering exciting potential for future research.

## Materials and methods

### Study population

Plasma samples collected from 54,265 UK Biobank participants at their baseline visit were measured using Olink Explore 3072 as a part of UKB-PPP (UK Biobank application number 65851)^52^. All participants provided informed consent. A large majority of the samples were randomly selected across the UK Biobank, and only those were used for the analysis presented here. In addition, the second delivery of data containing the last 1,462 proteins had an issue for the participants from batch 8. Unless specified otherwise, these participants were excluded from the analysis, resulting in the number of participants decreasing to 45,174.

### GWAS analysis

GWAS analysis was performed using the UK Biobank Research Analysis Platform (UKB RAP)^53^. The previously created clusters were used to extract phenotypic information. The genomic data used were array data (field 22418) and imputed data (field 21008). SNPs with high deletion rates, low secondary allele frequencies (MAFs), deviations from the Hardy-Weinberg equilibrium (HWE) and individuals with high genotype deletion rates were filtered out. The HWE exact test p value for the variant was greater than 10^− 15^, and the missing call rates for the variant and sample did not exceed 0.1. In the QC of the array data, the MAF was greater than 0.01, and the minor allele count (MAC) was greater than 100. The qc values of the imputed data were greater than 0.0001 and 10, respectively. Age and sex were regarded as covariates for the GWAS process. The steps of candidate gene mining included linkage disequilibrium (LD) clumping analysis and gene annotation.

### Health outcome coding (diagnostic codes)

The health outcomes in the UK Biobank are defined by the International Classification of Diseases (ICD-10) and are divided into 22 disease chapters covering primary care, hospital inpatient data, etiology, location, pathology and clinical manifestations. Symptoms and signs, classification, and onset times of acute and chronic diseases were recorded. Our analysis included 237 grouped 3-character ICD10s and 1725 3-character ICD10s (field 41270).

### Cluster construction

Two different methods were used to create the different clusters used in this study. The clusters named A1–A37 were built using the DIRAM method described below, and the clusters named B1–B18 were built using the DIRCOD method described below.

DIRAM method. Variables having identical or similar missing values were grouped together. Proteins with missing values for each group were discarded. The dimensionality of each of these groups was reduced to 2 dimensions using the scikit-learn implementation of the UMAP algorithm with default parameters except for the random seed. The resulting projection using 60 different random seeds (from 1 to 60) for each group was visualized to ensure that the project used was not the result of the algorithm being trapped in a local minimum. The clusters were then detected using the scikit-learn implementation of the DBSCAN algorithm, with the parameters min_samples = 10 and eps = 0.1, with the condition that the size of a cluster should be between 100 and 20,000 participants. Finally, the clusters issued from different groups were merged together if the list of participants included was similar, meaning that the difference between the expected distribution under the hypothesis that the probability of belonging to either cluster was independent and the observed distribution was greater than 40%.

DIRCOD method. The full dataset, with 45,174 participants, was used as a data object in scanpy^54^. The missing values were imputed using the scanpy built-in preprocessing function nearest neighbors using the parameter (n_neighbors = 15). The resulting data frame was subjected to the Leiden community detection algorithm implemented in scanpy using default parameters. The resulting communities were characterized using nonimputed data, and proteins with different distributions in any of the clusters were selected. A dataframe using only the selected proteins was subjected to the same Leiden algorithm. The resulting communities were characterized again using nonimputed data, and proteins with different distributions were selected. This iteration of Leiden selection of proteins was repeated 20 times. Participants with similar patterns of community assignment were grouped together into the same cluster.

### Definition of high concentration and low concentration of protein

In this study, we used the following definition for proteins with different concentrations (see Fig. 1): two proteins were at different concentrations if the top 3 quartiles of the distribution of the high concentration were higher than the highest quartile of the other population. Alternatively, a protein was considered to have a low concentration distribution if the bottom 3 quartiles of the distribution were lower than the lowest quartile of the other population.

### Cluster recreation

To recreate the different clusters, we looked at the values of the differently distributed proteins inside the group of clusters of interest. We then used the values corresponding to the different percentiles for each protein to apply a filter to the whole population and reported the odds ratio of the disease in the selected population. We performed the same analysis but omitted one different protein each time to determine the contribution of that specific protein.

### PCA

PCA was performed using the scikit-learn implementation of the PCA solver. The PCA transformation was fitted using the participants present in the clusters of interest in the first step. The participants in these clusters were subsequently binned into different groups of the same size depending on their value on the first dimension of the PCA, and the prevalence of the disease was subsequently calculated for each bin (see Fig. 4a and c). The same transformation was then applied to the whole population and reported separately (see Fig. 4b and d). A linear regression was fitted through all the data points except the lowest and the highest points due to the noise associated with these points.

### Correlation analysis

Proteins present in all considered clusters or in all but one of the considered clusters were used for this analysis. Proteins with a correlation of 0.8 for any other protein were kept; the others were discarded. Two correlation matrices were constructed for these proteins, the first one (A) using the values inside the group of clusters and the second one (B) using all the other participants. The result presented in this study is the matrix resulting from the difference between these two matrices (A–B) (see Fig. 5a).

### Statistical analysis and data manipulation

Unless stated otherwise, all the statistical analyses were performed using the Python language in the Jupyter environment, and all the p values reported have been adjusted for multiple testing with the Bonferroni correction. The following versions of the Python libraries were used: Python: 3.9.23; pandas: 2.3.0; numpy: 1.24.4; scipy: 1.13.1; sklearn: 1.6.1; matplotlib: 3.8.4; seaborn: 0.13.2; forestplot: 0.4.1; gwaslab: 3.6.8; and REGENIE: 2.0.0. Unless stated otherwise, all data analyses used the nonimputed data to avoid any bias that could have occurred during the imputation. Notebooks containing the different codes used in this study are available at https://github.com/Elvisbernard/unsupervised-learning-on-proteomics.

## Supplementary Information

Below is the link to the electronic supplementary material.

Supplementary Material 1

Supplementary Material 2

Supplementary Material 3

Supplementary Material 4

Supplementary Material 5

Supplementary Material 6

Supplementary Material 7

Supplementary Material 8

## Data Availability

The data used in the present study are available from the UK Biobank with restrictions applied. Data were used under license and are thus not publicly available. Access to the UK Biobank data can be requested through a standard protocol ( [https://www.ukbiobank.ac.uk/register-apply/](https:/www.ukbiobank.ac.uk/register-apply) ).
