# Supplementary material for: Unsupervised learning reveals novel disease-associated proteins in high-dimensional human proteomic data
Source: Sci Rep. 2026 Feb 22;16:10185. doi: 10.1038/s41598-026-41385-7 (PMC13022487; doi:10.1038/s41598-026-41385-7)
Supplement: Supplementary file 8 — Supplementary Material 8 [file 41598_2026_41385_MOESM8_ESM.docx]

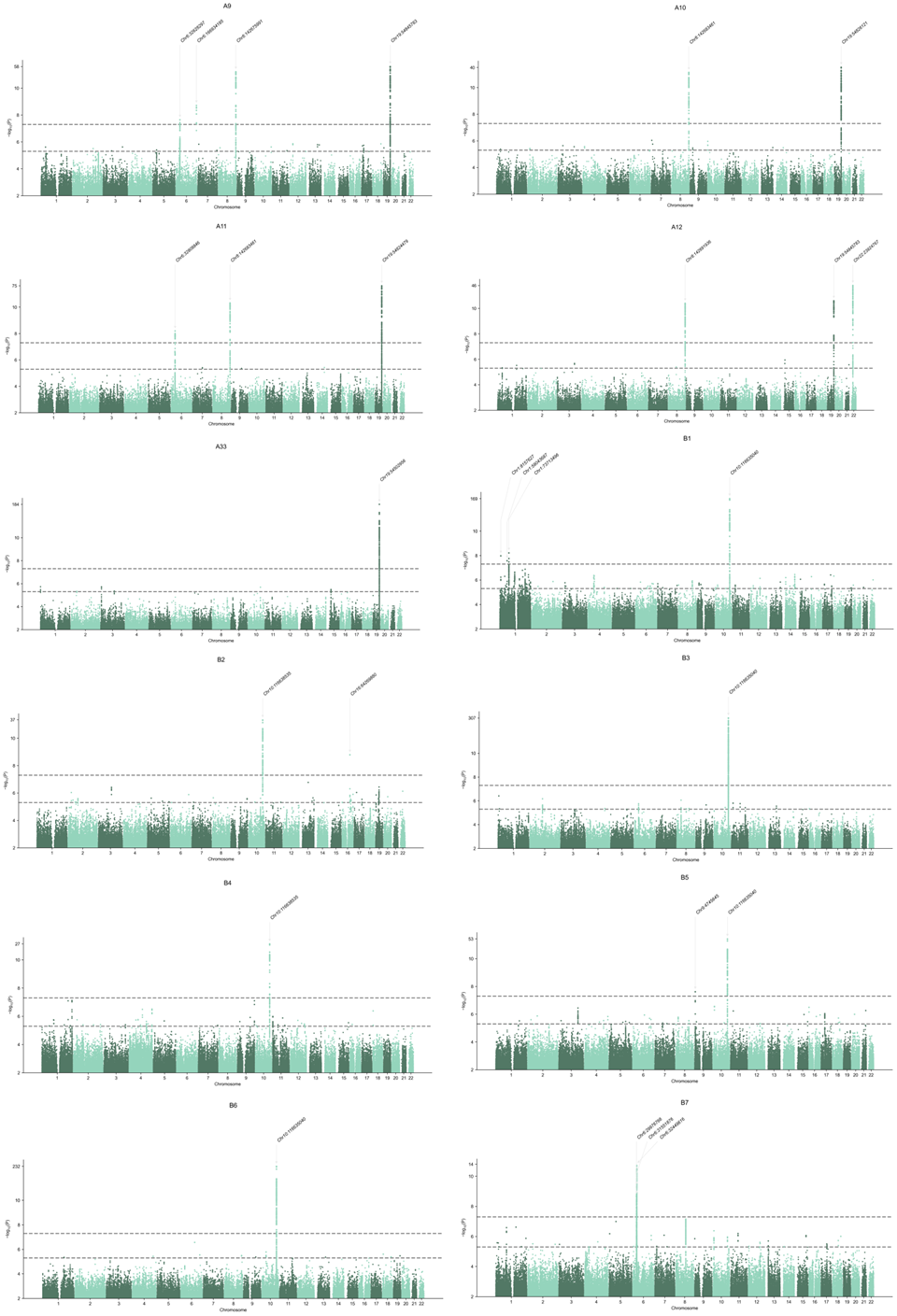


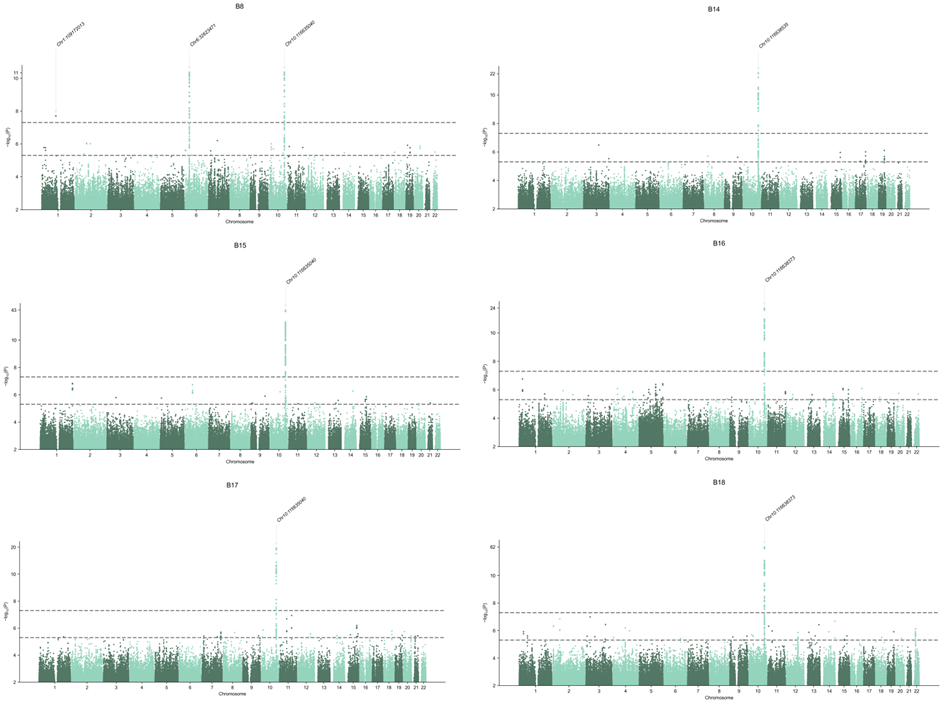


Figure S1:

**Manhattan plot of the GWAS for the most significant clusters**.


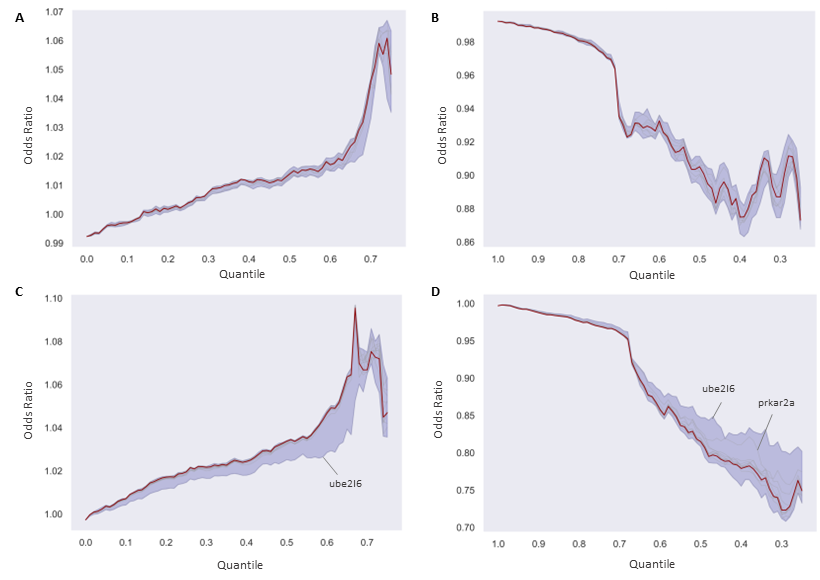


Figure S2:

**Alternative recreation of the clusters in the case of hypertension.** A and B: Hypertension with only cluster B15. C and D: Hypertension with the addition of cluster A7. The red line represents the odds ratio of the cluster when all the proteins were used; the green line represents the odds ratio of the cluster when all but one protein were used.
